# Characterization of patients with Duchenne muscular dystrophy across previously developed health states

**DOI:** 10.1371/journal.pone.0307118

**Published:** 2024-10-30

**Authors:** Francesco Muntoni, Nathalie Goemans, Nate Posner, James Signorovitch, Michaela Johnson, Chujun He, Patricia Dorling, Katherine Beaverson, Jose Alvir, Matthias Mahn, Susan J. Ward, Craig M. McDonald, Eugenio Mercuri, the PRO-DMD-01 study, the Imaging DMD study, iMDEX, the collaborative Trajectory Analysis Project (cTAP)

**Affiliations:** 1 Dubowitz Neuromuscular Centre, Great Ormond Street Institute of Child Health, London, United Kingdom; 2 University Hospitals Leuven, Child Neurology, Leuven, Belgium; 3 Pfizer, Inc., New York, NY, United States of America; 4 Analysis Group, Inc., Boston, MA, United States of America; 5 collaborative Trajectory Analysis Project (cTAP), Cambridge, MA, United States of America; 6 Chiesi USA, Inc. Boston, MA, United States of America; 7 Department of Physical Medicine and Rehabilitation, University of California, Davis, Sacramento, CA, United States of America; 8 Pediatric Neurology, Catholic University, Rome, Italy; 9 Centro Clinico NeMO, Fondazione Policlinico Gemelli IRCCS, Rome, Italy; Fondazione Policlinico Universitario Gemelli IRCCS, ITALY

## Abstract

Project HERCULES has developed a natural history model (NHM) of disease progression in Duchenne muscular dystrophy (DMD) that comprises eight ordered health states (two ambulatory states, one transfer state indicating increased caregiver burden in which patients cannot walk/run 10m or rise from floor but can still support their own weight, and five non-ambulatory states). The current study used data from nine sources (clinical trial placebo arms, one real-world dataset, and three natural history datasets) to further characterize patients with DMD according to these health states. The study included 1,173 patients across 5,306‬ visits. Patients were on average older and exhibited worse ambulatory, pulmonary, upper limb, and cardiac functions with each successive health state. Mean±SE ages increased monotonically across health states, starting with 8.47±0.07 for early ambulatory, 10.86±0.13 for late ambulatory, 11.65±0.35 for transfer state, and ranging from 13.17±0.32 to 16.84±0.37 for the non-ambulatory states. North Star Ambulatory Assessment (NSAA) total score, which measures motor function and ranges from 34 (best) to 0 (worst), was 23.7 (interquartile range [IQR]: 20–30) for early ambulatory patients, 12.7 (IQR: 9–16) for late ambulatory patients, and 3.9 (IQR: 2–5) for transfer patients. Pulmonary function as measured by mean±SE of forced vital capacity percent predicted (FVC%p) was 94.5±0.8 for early ambulatory, 89.1±1.4 for late ambulatory, and 80.2±2.8 for transfer states, and decreased from 77.2±1.7 to 20.6±1.6 across the five non-ambulatory health states. In summary, these findings further characterize health states and their interpretation in economic modeling and decision-making in DMD management.

## Introduction

Duchenne muscular dystrophy (DMD) is a rare, X-linked disorder, with a global prevalence estimated at 2.8 per 100,000 in the general population [1, 2]. DMD is caused by mutations in the dystrophin gene that result in progressive muscle weakness and degeneration beginning in early childhood [3]. Even with active monitoring and management of symptoms and comorbidities, the median life expectancy is 22–40 years [4, 5], with cardiac and respiratory failure as the main causes of death [6].

The natural history of DMD is marked by progressive declines and then losses in muscle function affecting ambulation, weight-bearing capacity, upper limb function and respiratory function, followed by cardiac failure [7]. As the life expectancy of patients with DMD increases with advances in diagnosis, care, and the availability of new treatments [8–11], it has been possible to delineate distinct stages of the disease, enabling comprehensive depictions of the disease trajectory. By developing models of the DMD’s natural history, a better understanding of patients’ healthcare needs at different stages of the disease can be achieved, thereby informing clinical and economic decision-making.

In this context, the Health Research Collaboration United in Leading Evidence Synthesis (HERCULES) project—an international collaboration between multiple stakeholders led by Duchenne UK—was initiated in order to develop evidence-based tools and collect data that can drive the therapeutic pipeline based on input from patients and caregivers as well as clinicians [12–14]. As one of the objectives, Project HERCULES has developed a natural history model (NHM) of DMD progression through eight distinct health states from early ambulatory to non-ambulatory that were defined using functional outcome measures commonly used in clinical trials and clinical practice [15]. The model included an intermediate transfer state in which weight bearing is possible but the patient can no longer rise from floor or walk/run for 10 m. In addition, non-ambulatory states were disaggregated to distinguish the different stages in which pulmonary function and upper-limb mobility are lost.

The present study aimed to further characterize the natural history of DMD health states by synthesizing data obtained from clinical trials, natural history studies, and real-world data. Specifically, the age, functional profiles, and steroid use were described among patients with DMD classified into health states consistent with those of Project HERCULES’ NHM [15]. Results from this study are expected to inform future modeling efforts and economic evaluations of treatments in DMD.

## Methods

### Data sources

This retrospective non-interventional study used data pooled from multiple sources that were accessed by the collaborative Trajectory Analysis Project (cTAP) in 2020–2021. These included phase 3 clinical trial placebo arm data from the PTC Therapeutics trial of ataluren (NCT01826487) [16, 17], Eli Lilly trial of tadalafil (NCT01865084) [18], and GlaxoSmithKline trial of drisapersen (NCT01254019) [19]. Additional data from real-world and natural history studies were obtained from the Leuven Neuromuscular Reference Center at the Universitaire Ziekenhuizen Leuven [20], PRO-DMD-01 prospective natural history study sponsored by BioMarin Pharmaceutical (data provided by CureDuchenne, a 501(3)c DMD patient foundation, NCT01753804) [21], North Star Clinic Network (NSUK) [22], iMDEX study funded by the French Muscular Dystrophy Association (NCT02780492) [23], and ImagingDMD study (NCT01484678) [24]. Detailed descriptions of these datasets have been reported in a previously published study [25].

The ethics committee at each institution (Universitaire Ziekenhuizen Leuven, PRO-DMD-01, and each participating center in iMDEX and ImagingDMD) approved the data sources. Written, informed consent or assent was obtained from participants or their caregiver before the study. For NSUK data, Caldicott Guardian regulations were followed including obtaining informed consent from patients’ guardian before entering patient information into the database and using only anonymous, deidentified data. The study adhered to the principles outlined in the Declaration of Helsinki.

### Classification of health states and sample selection

Project HERCULES developed an original model of the natural history of DMD based on input from clinicians, patients, and caregivers with consideration of data elements available in real-world data sources, natural history studies, and clinical trial data [14]. The health states map the progression of DMD through two ambulatory stages, one transfer stage, and five non-ambulatory stages defined based on functional performance and other measures: timed function tests (TFT), NSAA item scores [26], FVC%p, Brooke score for upper extremity ability, use of ventilation (none, night-time ventilation, and full-time ventilation), and hand-to-mouth function (HTMF).

**Fig 1:**
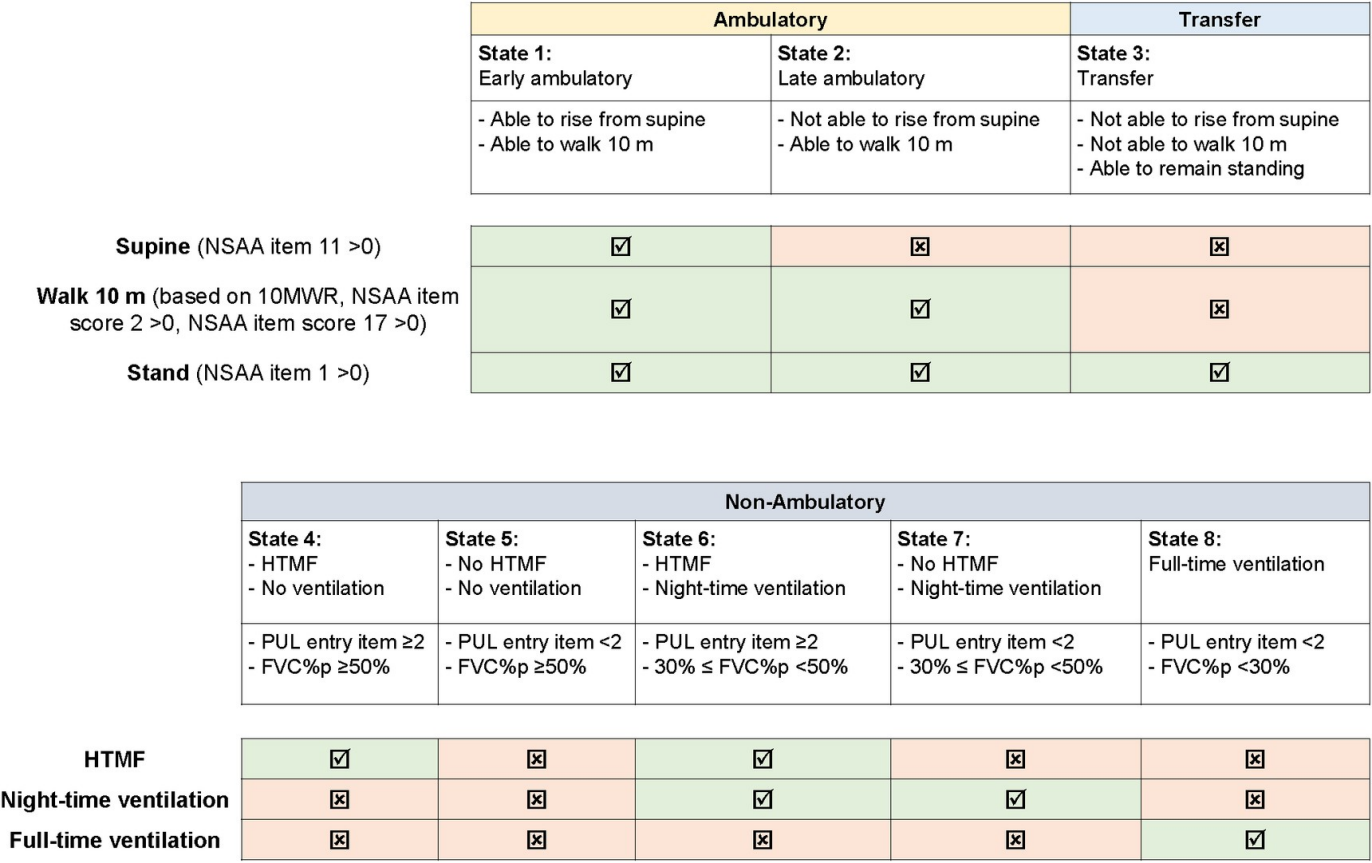
Definition of health states. Abbreviations: FVC%p, forced vital capacity percent predicted; HTMF, hand-to-mouth function; NSAA, North Star Ambulatory Assessment; PUL, Performance of Upper Limb.

The eight health states used in this study were defined to match those of Project HERCULES’ NHM as closely as possible, and were modified to accommodate most commonly available data elements in the data sources used in the current study (**Fig 1**) [14]. As a progressive disorder, different performance scales are used in different stages of DMD. By design, NSAA is mostly used in ambulatory stages while performance of the upper limb [PUL] score is used in non-ambulatory states. More specifically, in the present study, the ambulatory and transfer states were defined based on ability to rise from the supine position, walk 10 meters, and remain standing, determined by the corresponding NSAA item 11, item 2, and item 1, respectively. However, a sensitivity analysis was conducted where the ability to walk 10 m was defined based on a combination of multiple outcome measures that can be used to assess walking function (the timed 10-m walk/run test [10MWR], NSAA walk item 2, and NSAA run item 17) to account for variation in availability of different ambulatory assessments across data sources. The non-ambulatory states were based on upper extremity function (as measured by the entry item of the PUL score version 1.2) and pulmonary function (as measured by FVC%p). Two clinicians with expertise in DMD, Dr. Edward Smith, MD (a Pediatric Neurologist and Professor at Duke University School of Medicine) and Dr. Michela Guglieri, MD (a Senior Clinical Lecturer and Consultant Neurologist at Newcastle University and Newcastle Hospitals NHS Foundation Trust), reviewed these modified health state definitions for appropriateness and feasibility with real-world data sources, natural history studies, and clinical trial data.

To maximize use of the available data, the study sample comprised all patient visits that could be classified into a health state.

### Statistical methods

Within each health state, summary statistics were calculated for demographic characteristics, steroid (deflazacort or prednisone) use, and DMD measures (NSAA total score, timed 4-stair climb, health utility index, PUL total score and components, FVC%p, and left ventricular ejection fraction [LVEF]) based on visits in that health state with available data on the measures of interest. If a patient had multiple visits in a given health state, all visits were included in the summaries so that the full distribution of ages and clinical profiles within each heath state was represented. Means and standard errors were calculated for continuous measures using generalized estimating equations (GEEs) with an exchangeable covariance structure to account for use of multiple visits from individual patients. For the sensitivity analysis assessing the alternate definition for ability to walk 10 m, descriptive statistics were replicated to assess whether the restriction to only use NSAA items to define the first three states would alter the health states characterization. Results for this sensitivity analysis, as well as additional statistics characterizing the age at the first visit per health state per patient, including quantiles, mean, standard deviation, skewness and kurtosis, are reported in the Supplementary Information. Counts and percentages were obtained for categorical measures.

Histograms of age and FVC%p were generated to assess differences in patient age and pulmonary function by health state. A histogram of steroid use by health state was generated to assess differences by health state. Similarly, LVEF was plotted for patients in the 25th, 50th, and 75th percentiles by health state.

## Results

### Demographic characteristics

The study included 1,173 patients with DMD across 5,306 visits (**Table 1**). Patients could contribute multiple visits within and across health states. Overall, the majority of visits were early ambulatory (N = 3,920) or late ambulatory (N = 1,019); visit counts for transfer and non-ambulatory states were relatively small, representing a total of N = 367 visits with the largest sample in the first non-ambulatory state (HTMF, no ventilation; N = 208).

**Table 1:** Summary of characteristics across health states^a^^–^^d^.

** **	**Early ambulatory**	**Late ambulatory**	**Transfer**	**HTMF, no ventilation**	**No HTMF, no ventilation**	**HTMF, night-time ventilation**	**No HTMF, night-time ventilation**	**Full-time ventilation**
** **	N = 3,920	N = 1,019	N = 60	N = 208	N = 24	N = 31	N = 24	N = 20
** **	(951 patients)	(403 patients)	(50 patients)	(82 patients)	(15 patients)	(17 patients)	(9 patients)	(10 patients)
**Demographics and medications**	** **	** **	** **	** **	** **	** **	** **	** **
Age (years)	8.47 ± 0.07	10.86 ± 0.13	11.65 ± 0.35	13.17 ± 0.32	14.33 ± 0.62	14.94 ± 0.68	16.44 ± 0.59	16.84 ± 0.37
Height (cm)	121.08 ± 0.35	131.39 ± 0.59	141.27 ± 2.27	143.56 ± 1.64	153.24 ± 4.29	151.03 ± 4.20	157.50 ± 3.79	164.62 ± 2.10
Weight (kg)	28.02 ± 0.30	36.69 ± 0.69	47.09 ± 2.60	48.76 ± 1.78	51.15 ± 3.85	56.24 ± 5.00	63.89 ± 4.83	60.88 ± 6.11
Race								
White	1,545 (85.55%)	585 (79.05%)	19 (95.00%)	177 (88.06%)	24 (100.00%)	30 (96.77%)	24 (100.00%)	17 (100.00%)
Asian	142 (7.86%)	41 (5.54%)	1 (5.00%)	6 (2.99%)	0 (0.00%)	0 (0.00%)	0 (0.00%)	0 (0.00%)
Black or African American	19 (1.05%)	23 (3.11%)	0 (0.00%)	1 (0.50%)	0 (0.00%)	0 (0.00%)	0 (0.00%)	0 (0.00%)
Other	100 (5.54%)	91 (12.30%)	0 (0.00%)	17 (8.46%)	0 (0.00%)	1 (3.23%)	0 (0.00%)	0 (0.00%)
Ethnicity								
Hispanic or Latino	219 (20.02%)	80 (18.82%)	0 (0.00%)	2 (40.00%)	0 (0.00%)	-	-	-
Not Hispanic or Latino	875 (79.98%)	345 (81.18%)	17 (100.00%)	3 (60.00%)	1 (100.00%)	-	-	-
Steroid use								
Deflazacort or Prednisone	3,217 (93.00%)	936 (97.70%)	48 (100.00%)	191 (91.83%)	19 (79.17%)	26 (83.87%)	16 (66.67%)	2 (10.00%)
On daily regimen	1,979 (66.70%)	628 (71.44%)	25 (58.14%)	134 (70.16%)	12 (63.16%)	17 (65.38%)	14 (87.50%)	2 (100.00%)
Not on steroids	242 (7.00%)	22 (2.30%)	0 (0.00%)	17 (8.17%)	5 (20.83%)	5 (16.13%)	8 (33.33%)	18 (90.00%)
**DMD measures**	** **	** **	** **	** **	** **	** **	** **	** **
NSAA total score	23.69 ± 0.19	12.71 ± 0.24	3.94 ± 0.27					
NSAA total score IQR	(20.00, 30.00)	(9.00, 16.00)	(2.00, 5.00)					
Timed 4-stair climb (seconds)	4.50 ± 0.13	13.18 ± 0.54	28.59 ± 1.42					
Health utility index	0.83 ± 0.02	0.77 ± 0.04	0.67 ± 0.13					
PUL								
Total score				61.30 ± 0.73	38.00 ± 1.85	53.58 ± 2.73	30.99 ± 3.02	21.91 ± 3.93
Entry question				4.55 ± 0.12	0.96 ± 0.04	3.43 ± 0.27	1.00 ± -	1.00 ± 0.00
HTMF				2.79 ± 0.05	0.89 ± 0.19	2.28 ± 0.18	0.31 ± 0.12	0.24 ± 0.06
Remove lid from container				0.93 ± 0.02	0.74 ± 0.11	0.90 ± 0.05	0.52 ± 0.13	0.13 ± 0.09
Push on the light				2.61 ± 0.05	2.10 ± 0.20	2.30 ± 0.17	1.50 ± 0.23	0.89 ± 0.27
FVC%p (%)	94.47 ± 0.77	89.13 ± 1.35	80.24 ± 2.83	77.20 ± 1.69	69.34 ± 4.55	42.34 ± 1.19	39.19 ± 1.27	20.57 ± 1.63
Left ventricular ejection fraction (%)	63.91 ± 0.37	61.85 ± 0.91	59.41 ± 2.34	56.87 ± 1.36	55.10 ± 4.55	59.07 ± 48.69	46.77 ± 4.91	47.70 ± 2.81

**Abbreviations:** DMD, Duchenne muscular dystrophy; FVC%p, forced vital capacity percent predicted; HTMF, hand-to-mouth function; NSAA, North Star Ambulatory Assessment; PUL, Performance of Upper Limb; IQR, Interquartile range.

Notes

[a] Data are presented as mean±SE or n (%). To account for correlations with multiple observations per patient, means and standard errors were estimated using generalized estimating equations (GEEs).

[b] Two outliers, one with height ≤11.8 cm and one with weight ≤3.3 kg, were removed from the calculations.

[c] Percentages were calculated from non-missing data.

[d] Gray cells correspond to unavailable data for a given health state.

**Fig 2:**
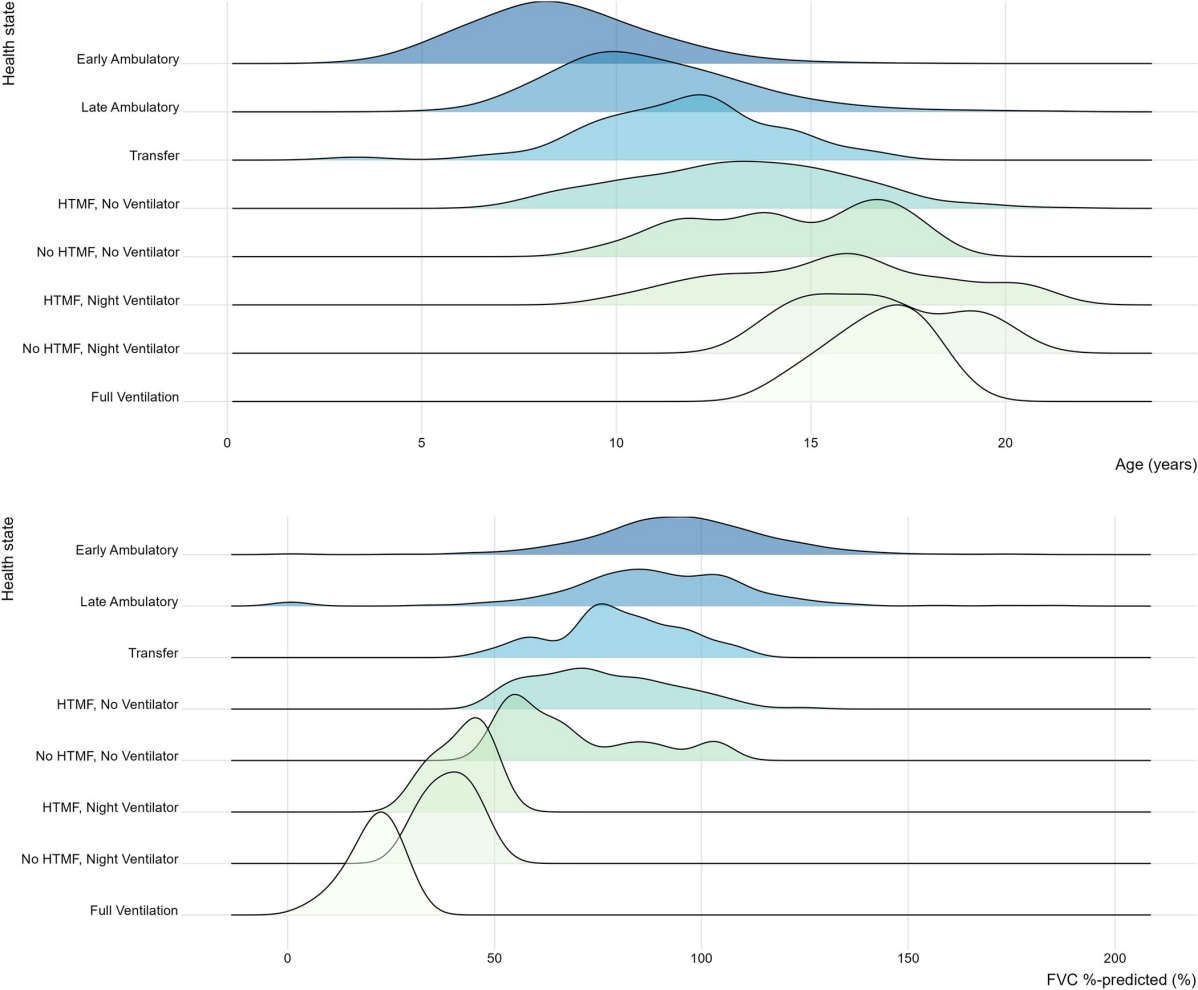
Distributions of patient ages (A) and levels of pulmonary function (B) across health states. Abbreviations: FVC, forced vital capacity; HTMF, hand-to-mouth function.

Patient age increased on average in later (i.e., more progressed) health states, notably with overlapping distributions across and broad heterogeneity within health states (**Fig 2** and **S1 Table**), from a mean±SE age of 8.47±0.07 years in the early ambulatory state where patients still have the ability to rise from the floor, walk and remain standing, 11.65±0.35 years in the transfer state where they can only stand, 13.17±0.32 years in the first state where they have lost ambulation (HTMF, no ventilation), to 16.84±0.37 years in the last health state. Statistics characterizing the ages at first visit per state show that excess kurtosis tends to be positive for the ambulatory and transfer states, indicating a heavier tail than the normal distribution, further highlighting the wide range of functional motor ability in early stages of the disease (**S2 Table**). For example, 1% of patients enter the late ambulatory state at or after 17.99 years of age. Conversely, excess kurtosis tends to be negative for non-ambulatory states, indicating few outliers, although these results may be influenced by the limited data available for these later disease stages.

The sensitivity analysis that defined the ability to walk 10 m based on several outcome measures (the timed 10MWR, NSAA walk item 2, and NSAA run item 17) as opposed to only NSAA item 2 resulted in slightly more visits captured in the ambulatory states (4,950 v. 4,939 visits) but fewer visits captured in the transfer state (39 v. 60 visits). While the relative impact of this alternative definition was most pronounced in the transfer state in terms of visit sample size, the characterization of this state in terms of age, height, weight and NSAA total score was essentially unchanged. Overall, the sample characteristics within each health state were consistent with those from the main analysis indicating alignment between definitions (**S3 Table**).

### Steroid use

**Fig 3:**
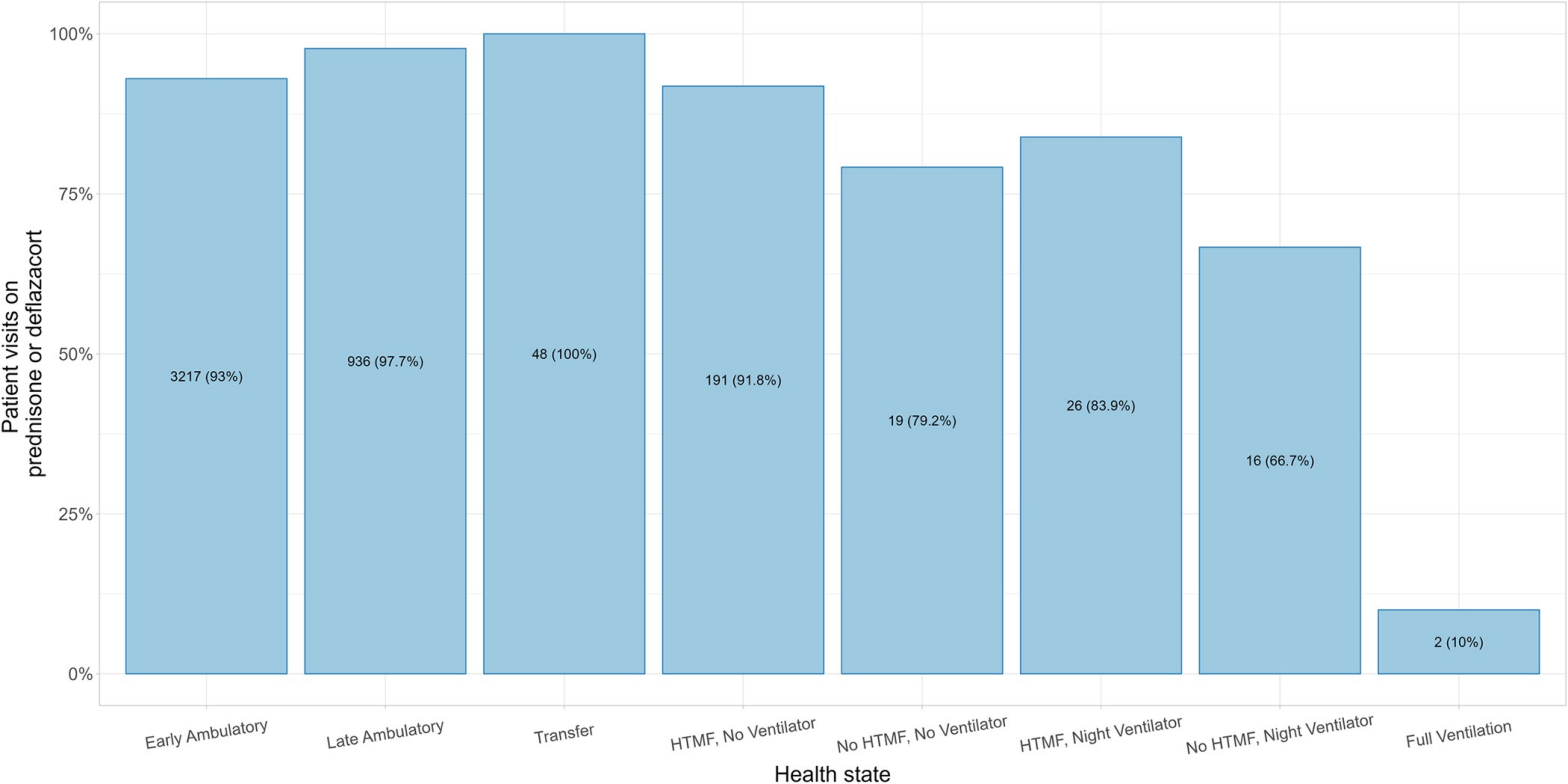
Steroid use across health states. Percents are based on the visits in each respective health state. Abbreviations: HTMF, hand-to-mouth function.

Compared with patients in States 5–8, those in States 1–4 were more likely to use steroids (deflazacort or prednisone) (**Table 1** and **Fig 3**). Across all health states, among those using steroids, the majority reported being on a daily regimen, with 66.70% for State 1 (early ambulatory), 71.44% for State 2 (late ambulatory), and 58.14% for State 3 (transfer) (**Table 1**).

### Functional outcomes

Patients in earlier health states scored better on multiple outcome measures. Ambulatory function declined on average across the progressively ordered ambulatory and transfer health states, as evidenced by decreasing average NSAA total score, health utility index, and longer timed 4-stair climb. In particular, average NSAA total score was 23.69±0.19 (interquartile range [IQR]: 20–30) for early ambulatory patients, 12.71±0.24 (IQR: 9–16) for late ambulatory patients, and 3.94±0.27 (IQR: 2–5) for transfer patients. In non-ambulatory states, PUL total score also decreased in later health states, reflecting worse functional outcomes, from 61.30±0.73 to 21.91±3.93 across the five non-ambulatory health states (**Table 1**).

**Fig 4:**
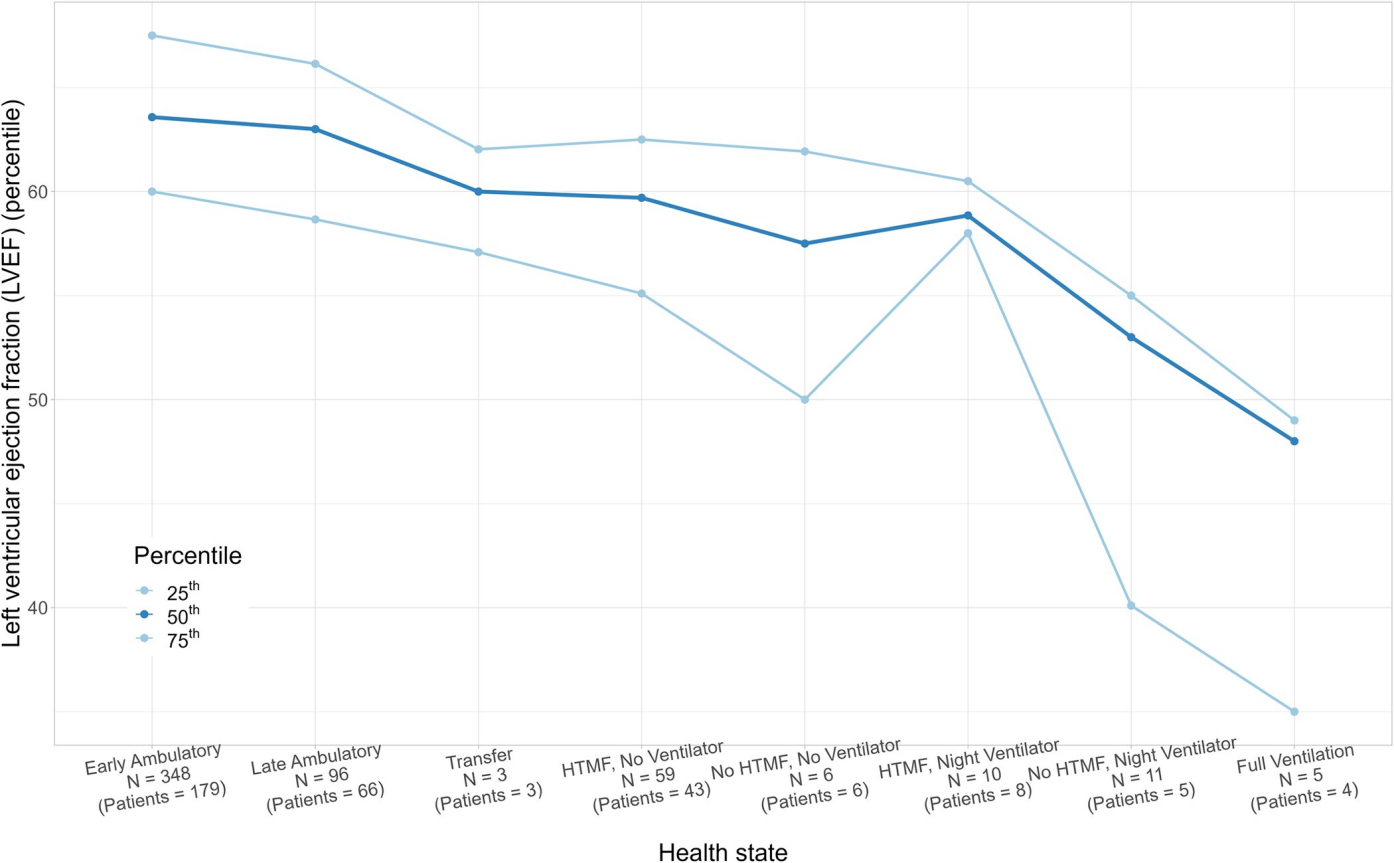
Left ventricular ejection fraction across health states. Sample sizes and patient counts reported per health state represent observations with available data for left ventricular ejection fraction. Percentiles are based on the visits in each respective health state. Abbreviations: HTMF, hand-to-mouth function.

Patients in ambulatory and transfer states had better pulmonary function as indicated by higher FVC%p, which averaged 94.47% in the early ambulatory state and decreased to 80.24% in the transfer state. In non-ambulatory states, FVC%p decreased uniformly from an average of 77.20% in State 4 (HTMF, no ventilation) to 20.57% in State 8 (full-time ventilation) (**Table 1** and **Fig 2**). Similar to pulmonary function, cardiac function was generally better in the ambulatory and transfer health states than in non-ambulatory states (**Table 1** and **Fig 4**), with LVEF ranging from an average of 59.41%–63.91% in ambulatory states and 46.77%–59.07% in non-ambulatory states.

## Discussion

The present study characterized patients with DMD according to the health states representing the natural history of DMD defined by Project HERCULES [14, 15]. The modified health states aligned with previously published literature. These health states that span the DMD disease spectrum are unique in terms of the combination of ambulatory function, upper limb function, and pulmonary FVC%p values associated with the use of night-time and full-time ventilation. Overall, functional outcomes including ambulatory, pulmonary, upper limb, and cardiac functions declined with each successive health state. These results provide a detailed representation of patients with DMD at each stage of disease course, including a transfer state which marks the key transition from ambulation to non-ambulation. Periods shortly before and after loss of ambulation in DMD have been recently characterized to facilitate broader inclusion of these patients in clinical studies [27].

The health state characterizations here were robust to changes in the functional outcome measures used to define the ability to walk 10 m. Namely, using only the NSAA walk item versus using a combination of the NSAA walk item, NSAA run item, and the 10MWR had a negligible impact on estimated mean and standard errors across a diversity of characteristics which included age, height, weight, and NSAA total score. Thus, the simpler definition using just the NSAA walk item may be more appropriate for future analyses of clinical trial data, where missing NSAA item scores are less likely given stringent protocols.

The loss of ambulation is the first major milestone in DMD. In the NHM, non-ambulation is modeled in States 4–8. However, patients first lose the ability to walk when they transition from State 2 (late ambulatory) to State 3 (transfer). This period (the loss of ambulation) typically occurs between the ages of 10 and 13 years, according to the clinical literature [28–35]. In the current study, the estimated mean age of patients was 11.7 years in the transfer state, which falls within this range, but is slightly higher than the mean age in State 3 estimated by Broomfield et al. (10.3 years) [15]. These differences may be attributable to assumptions underlying projections of NHM transition probabilities as discussed in Broomfield et al [15], where an elicitation exercise was used due to paucity of data, which the authors acknowledge may affect the estimates associated with transitions into and out of health state 3. Future research should explore further alignment of NH data and transition probabilities using real world data from larger populations of DMD patients.

A later major milestone in DMD is the use of mechanical ventilation, which starts at the age of approximately 20 years according to the clinical literature [31, 35–38]. The start of ventilation in the NHM is modeled in the transition from State 4 to 6, or from State 5 to 7 (transitions to States 5 and 6 do not happen sequentially, since there is no fixed order in which start of ventilation or loss of HTMF can occur) [15]. The sample mean age at States 6 and 7 were 14.9 and 16.4 years, respectively. These estimates overlap with Broomfield et al.’s [15], but are different from those in the literature (19–26 years [28, 39, 40]), possibly reflecting some attrition bias as patients with worsening condition left the study samples. It may also reflect differences in the frequency of steroid use across the study samples as corticosteroids have been associated with prolonged respiratory function [41]. Still, this study confirmed that pulmonary function (FVC%p) showed a gradual and sequential deterioration of pulmonary function from State 1 to 8. There was also a decrease in cardiac function (LVEF), consistent with the increasing prevalence of cardiomyopathy with DMD progression [28, 35], with a steep decline starting at State 6. Further studies could refine these later health state characterizations by obtaining larger samples for non-ambulatory states, either through data from more patients or by adjusting the aggregation of non-ambulatory health state definitions (e.g., grouping into three states rather than five).

In the present cohort, the loss of one of the most important aspects of upper limb function—indicated by the loss of HTMF [42] deduced from the PUL entry item [43, 44]—occurred in (non-ambulatory) State 5, in which the mean age was 14.3 years. Similarly, the median age at loss of HTMF reported in other studies is approximately 15 years [29, 45]. Although HTMF may be initially retained even as lung function begins to deteriorate (State 6), it decreases progressively in non-ambulatory patients [46]. Previous research from the Cooperative International Neuromuscular Research Group (CINRG) demonstrated a strong linear relationship between upper limb function (through Brooke scores) and FVC%p values in DMD patients [47], supporting the incorporation of both HTMF and pulmonary function values associated with ventilatory use in non-ambulatory patients. CINRG’s previously described natural history framework used timed function tests and upper limb function (using Brooke scores) [29]. Thus, the transition from late ambulatory to transfer to early non-ambulatory states represents an important period during which enhanced respiratory surveillance and respiratory interventions are needed. This observation is particularly important for those DMD patients with intellectual disability or behavioral comorbidities who might not comply with the instructions for performing respiratory function test accurately.

### Strengths and limitations

A strength of this study was the DMD patient population pooled from nine different data sources, which provided a large sample of patients with this rare disease. However, due to the short-term follow up times of most data sources, this study focused on estimating aggregate cross-sectional statistics describing the states. As a consequence, a limitation of this study was that the various data sources contributed varying numbers of patients across different disease health states, which may have impacted patterns of steroid use and other variables. There were other limitations that warrant mention. Sample sizes for the transfer and non-ambulatory states and for older patients (>18 years) were small. Additionally, many clinics and data sources do not assess NSAA scores when patients cannot stand, which may have resulted in underrepresentation of patients with documented NSAA stand scores equal to 0 and underrepresentation of the transfers health state.

## Conclusion

Average levels of function including ambulatory motor, pulmonary, upper-limb, and cardiac functions showed progressive worsening through the eight health states that have been proposed for DMD progression. These data provide additional evidence that the studied health states provide an informative framework for categorizing stages of disease progression in DMD that accords with understanding of natural history. These health states can provide a helpful starting point for health economic evaluations in DMD, particularly during the ambulatory stages of the disease, when complemented by data on quality of life and rates of progression across health states.

## Supporting information

S1 Table Age distribution quantiles across health states for all visits. (DOCX)

S2 Table Statistics for ages at first visits per health state per patient. (DOCX)

S3 Table Summary of characteristics across health states in sensitivity analysis. (DOCX)

## References

[1] Crisafulli S, Sultana J, Fontana A, Salvo F, Messina S, Trifirò G. Global epidemiology of Duchenne muscular dystrophy: an updated systematic review and meta-analysis. Orphanet J Rare Dis. 2020;15(1):141. Epub 20200605. doi: 10.1186/s13023-020-01430-8 ; PubMed Central PMCID: PMC7275323. 32503598 PMC7275323

[2] Salari N, Fatahi B, Valipour E, Kazeminia M, Fatahian R, Kiaei A, et al. Global prevalence of Duchenne and Becker muscular dystrophy: a systematic review and meta-analysis. J Orthop Surg Res. 2022;17(1):96. Epub 20220215. doi: 10.1186/s13018-022-02996-8 ; PubMed Central PMCID: PMC8848641. 35168641 PMC8848641

[3] Duan D, Goemans N, Takeda S, Mercuri E, Aartsma-Rus A. Duchenne muscular dystrophy. Nat Rev Dis Primers. 2021;7(1):13. Epub 20210218. doi: 10.1038/s41572-021-00248-3 . 33602943 PMC10557455

[4] Broomfield J, Hill M, Guglieri M, Crowther M, Abrams K. Life Expectancy in Duchenne Muscular Dystrophy: Reproduced Individual Patient Data Meta-analysis. Neurology. 2021;97(23):e2304–e14. Epub 20211013. doi: 10.1212/WNL.0000000000012910 ; PubMed Central PMCID: PMC8665435. 34645707 PMC8665435

[5] Landfeldt E, Thompson R, Sejersen T, McMillan HJ, Kirschner J, Lochmüller H. Life expectancy at birth in Duchenne muscular dystrophy: a systematic review and meta-analysis. Eur J Epidemiol. 2020;35(7):643–53. Epub 20200227. doi: 10.1007/s10654-020-00613-8 ; PubMed Central PMCID: PMC7387367. 32107739 PMC7387367

[6] Wahlgren L, Kroksmark AK, Tulinius M, Sofou K. One in five patients with Duchenne muscular dystrophy dies from other causes than cardiac or respiratory failure. Eur J Epidemiol. 2022;37(2):147–56. Epub 20211121. doi: 10.1007/s10654-021-00819-4 ; PubMed Central PMCID: PMC8960570. 34802091 PMC8960570

[7] Bushby K, Finkel R, Birnkrant DJ, Case LE, Clemens PR, Cripe L, et al. Diagnosis and management of Duchenne muscular dystrophy, part 1: diagnosis, and pharmacological and psychosocial management. Lancet Neurol. 2010;9(1):77–93. Epub 20091127. doi: 10.1016/S1474-4422(09)70271-6 . 19945913

[8] Birnkrant DJ, Bushby K, Bann CM, Alman BA, Apkon SD, Blackwell A, et al. Diagnosis and management of Duchenne muscular dystrophy, part 2: respiratory, cardiac, bone health, and orthopaedic management. Lancet Neurol. 2018;17(4):347–61. Epub 20180203. doi: 10.1016/S1474-4422(18)30025-5 ; PubMed Central PMCID: PMC5889091. 29395990 PMC5889091

[9] Birnkrant DJ, Bushby K, Bann CM, Apkon SD, Blackwell A, Brumbaugh D, et al. Diagnosis and management of Duchenne muscular dystrophy, part 1: diagnosis, and neuromuscular, rehabilitation, endocrine, and gastrointestinal and nutritional management. Lancet Neurol. 2018;17(3):251–67. Epub 20180203. doi: 10.1016/S1474-4422(18)30024-3 ; PubMed Central PMCID: PMC5869704. 29395989 PMC5869704

[10] Birnkrant DJ, Bushby K, Bann CM, Apkon SD, Blackwell A, Colvin MK, et al. Diagnosis and management of Duchenne muscular dystrophy, part 3: primary care, emergency management, psychosocial care, and transitions of care across the lifespan. Lancet Neurol. 2018;17(5):445–55. Epub 20180202. doi: 10.1016/S1474-4422(18)30026-7 ; PubMed Central PMCID: PMC5902408. 29398641 PMC5902408

[11] US Food and Drug Administration. FDA Approves First Gene Therapy for Treatment of Certain Patients with Duchenne Muscular Dystrophy. June 23, 2023. Available at: https://www.fda.gov/news-events/press-announcements/fda-approves-first-gene-therapy-treatment-certain-patients-duchenne-muscular-dystrophy. Accessed July 2, 2023.

[12] Crossley E, Chandler F, Godfrey J, Abrams K, et al. PROJECT HERCULES: Overcoming the Health Technology Assessment hurdle through patient led collaboration. TREAT-NMD International Conference 2019. Leiden, The Netherlands; December 9–11, 2019. Available at: https://hercules.duchenneuk.org/publications/. Accessed July 5, 2023

[13] Abrams K, Carlton J, Chandler F, Crossley E, Crowther M, Ghosh S, et al. Project HERCULES: Could international collaboration on value assessment change clinical practice? EP. 41. Neuromuscular Disorders. 2019;29:S112–S3.

[14] Broomfield J, Hill M, Crowther M, Larkindale J, Guglieri M, Godfrey J, editors. Project HERCULES: The challenges of estimating multi-state model transitions in rare diseases: creating a natural history model for Duchenne muscular dystrophy. Poster no. 173. European Conference on Rare Diseases and Orphan Products May 14–15, 2020 (Virtual) Available at: https://herculesduchenneukorg/publications/ Accessed July 5, 2023; 2020.

[15] Broomfield J, Hill M, Chandler F, Crowther MJ, Godfrey J, Guglieri M, et al. Developing a Natural History Model for Duchenne Muscular Dystrophy. Pharmacoecon Open. 2024;8(1):79–89. Epub 20231129. doi: 10.1007/s41669-023-00450-x ; PubMed Central PMCID: PMC10781931. 38019449 PMC10781931

[16] NCT01826487. Phase 3 Study of Ataluren in Participants With Nonsense Mutation Duchenne Muscular Dystrophy (nmDMD).

[17] NCT01826487. An Extension Study of Ataluren (PTC124) in Participants With Nonsense Mutation Dystrophinopathy.

[18] NCT01865084. A Study of Tadalafil for Duchenne Muscular Dystrophy.

[19] NCT01254019. A Clinical Study to Assess the Efficacy and Safety of GSK2402968 in Subjects With Duchenne Muscular Dystrophy.

[20] European Reference Network for Rare Neuromuscular Diseases. University Hospitals Leuven, Neuromuscular Reference Centre. Available at: https://ern-euro-nmd.eu/healthcare-provider/university-hospitals-leuven/. Accessed July 5, 2023.

[21] NCT01753804. A Prospective Natural History Study of Progression of Subjects With Duchenne Muscular Dystrophy.

[22] NorthStar Clinical Network. UK National Neuromuscular Database. Available at: https://www.northstardmd.com/. Accessed July 5, 2023.

[23] NCT02780492. Outcome Measures in Duchenne Muscular Dystrophy: A Natural History Study.

[24] NCT01484678. Magnetic Resonance Imaging and Biomarkers for Muscular Dystrophy.

[25] Muntoni F, Signorovitch J, Sajeev G, Goemans N, Wong B, Tian C, et al. Real-world and natural history data for drug evaluation in Duchenne muscular dystrophy: suitability of the North Star Ambulatory Assessment for comparisons with external controls. Neuromuscular Disorders. 2022;32(4):271–83. doi: 10.1016/j.nmd.2022.02.009 35396092

[26] Scott E, Eagle M, Mayhew A, Freeman J, Main M, Sheehan J, et al. Development of a Functional Assessment Scale for Ambulatory Boys with Duchenne Muscular Dystrophy. Physiotherapy Research International. 2012;17(2):101–9. doi: 10.1002/pri.520 21954141

[27] McDonald CM, Signorovitch J, Mercuri E, Niks EH, Wong B, Fillbrunn M, et al. Functional trajectories before and after loss of ambulation in Duchenne muscular dystrophy and implications for clinical trials. [Manuscript under review]. 2023. 10.1371/journal.pone.0304099 PMC11146704 38829874

[28] Koeks Z, Bladen CL, Salgado D, van Zwet E, Pogoryelova O, McMacken G, et al. Clinical Outcomes in Duchenne Muscular Dystrophy: A Study of 5345 Patients from the TREAT-NMD DMD Global Database. J Neuromuscul Dis. 2017;4(4):293–306. doi: 10.3233/JND-170280 ; PubMed Central PMCID: PMC5701764. 29125504 PMC5701764

[29] McDonald CM, Henricson EK, Abresch RT, Duong T, Joyce NC, Hu F, et al. Long-term effects of glucocorticoids on function, quality of life, and survival in patients with Duchenne muscular dystrophy: a prospective cohort study. Lancet. 2018;391(10119):451–61. Epub 20171122. doi: 10.1016/S0140-6736(17)32160-8 . 29174484

[30] Qian C, Klimchak A, Szabo S, Popoff E, Iannaccone S, Gooch K. Characterizing the natural history of Duchenne muscular dystrophy in the United States in real-world commercial and Medicaid data—PMS93 Value in Health. 2020;23:S232.

[31] Ryder S, Leadley RM, Armstrong N, Westwood M, de Kock S, Butt T, et al. The burden, epidemiology, costs and treatment for Duchenne muscular dystrophy: an evidence review. Orphanet J Rare Dis. 2017;12(1):79. Epub 20170426. doi: 10.1186/s13023-017-0631-3 ; PubMed Central PMCID: PMC5405509. 28446219 PMC5405509

[32] Szabo SM, Klimchak AC, Qian C, Iannaccone S, Popoff E, Gooch KL. Characterizing the Occurrence of Key Clinical Milestones in Duchenne Muscular Dystrophy in the United States Using Real-World Data. J Neuromuscul Dis. 2022;9(6):689–99. doi: 10.3233/JND-220816 ; PubMed Central PMCID: PMC9697036. 36245384 PMC9697036

[33] Ricotti V, Ridout DA, Pane M, Main M, Mayhew A, Mercuri E, et al. The NorthStar Ambulatory Assessment in Duchenne muscular dystrophy: considerations for the design of clinical trials. J Neurol Neurosurg Psychiatry. 2016;87(2):149–55. Epub 20150302. doi: 10.1136/jnnp-2014-309405 ; PubMed Central PMCID: PMC4752678. 25733532 PMC4752678

[34] Bello L, Morgenroth LP, Gordish-Dressman H, Hoffman EP, McDonald CM, Cirak S. DMD genotypes and loss of ambulation in the CINRG Duchenne Natural History Study. Neurology. 2016;87(4):401–9. Epub 20160624. doi: 10.1212/WNL.0000000000002891 ; PubMed Central PMCID: PMC4977110. 27343068 PMC4977110

[35] Szabo SM, Salhany RM, Deighton A, Harwood M, Mah J, Gooch KL. The clinical course of Duchenne muscular dystrophy in the corticosteroid treatment era: a systematic literature review. Orphanet J Rare Dis. 2021;16(1):237. Epub 20210522. doi: 10.1186/s13023-021-01862-w ; PubMed Central PMCID: PMC8141220. 34022943 PMC8141220

[36] Kieny P, Chollet S, Delalande P, Le Fort M, Magot A, Pereon Y, et al. Evolution of life expectancy of patients with Duchenne muscular dystrophy at AFM Yolaine de Kepper centre between 1981 and 2011. Ann Phys Rehabil Med. 2013;56(6):443–54. Epub 20130624. doi: 10.1016/j.rehab.2013.06.002 . 23876223

[37] Kohler M, Clarenbach CF, Bahler C, Brack T, Russi EW, Bloch KE. Disability and survival in Duchenne muscular dystrophy. J Neurol Neurosurg Psychiatry. 2009;80(3):320–5. Epub 20080819. doi: 10.1136/jnnp.2007.141721 . 18713792

[38] Rall S, Grimm T. Survival in Duchenne muscular dystrophy. Acta Myol. 2012;31(2):117–20. ; PubMed Central PMCID: PMC3476855. 23097602 PMC3476855

[39] Kelley EF, Cross TJ, Snyder EM, McDonald CM, Hoffman EP, Bello L. Influence of β(2) adrenergic receptor genotype on risk of nocturnal ventilation in patients with Duchenne muscular dystrophy. Respir Res. 2019;20(1):221. Epub 20191016. doi: 10.1186/s12931-019-1200-1 ; PubMed Central PMCID: PMC6796481. 31619245 PMC6796481

[40] LoMauro A, Romei M, Gandossini S, Pascuzzo R, Vantini S, D’Angelo MG, et al. Evolution of respiratory function in Duchenne muscular dystrophy from childhood to adulthood. Eur Respir J. 2018;51(2). Epub 20180207. doi: 10.1183/13993003.01418–2017 . 29437939

[41] Trucco F, Domingos JP, Tay CG, Ridout D, Maresh K, Munot P, et al. Cardiorespiratory Progression Over 5 Years and Role of Corticosteroids in Duchenne Muscular Dystrophy: A Single-Site Retrospective Longitudinal Study. Chest. 2020;158(4):1606–16. doi: 10.1016/j.chest.2020.04.043 32387519

[42] Mazzone ES, Vasco G, Palermo C, Bianco F, Galluccio C, Ricotti V, et al. A critical review of functional assessment tools for upper limbs in Duchenne muscular dystrophy. Dev Med Child Neurol. 2012;54(10):879–85. Epub 20120619. doi: 10.1111/j.1469-8749.2012.04345.x . 22713125

[43] Mayhew A, Mazzone ES, Eagle M, Duong T, Ash M, Decostre V, et al. Development of the Performance of the Upper Limb module for Duchenne muscular dystrophy. Dev Med Child Neurol. 2013;55(11):1038–45. Epub 20130801. doi: 10.1111/dmcn.12213 . 23902233

[44] Mayhew AG, Coratti G, Mazzone ES, Klingels K, James M, Pane M, et al. Performance of Upper Limb module for Duchenne muscular dystrophy. Dev Med Child Neurol. 2020;62(5):633–9. Epub 20190919. doi: 10.1111/dmcn.14361 . 31538331

[45] Naarding KJ, Janssen M, Boon RD, Bank PJM, Matthew RP, Kurillo G, et al. The Black Box of Technological Outcome Measures: An Example in Duchenne Muscular Dystrophy. J Neuromuscul Dis. 2022;9(4):555–69. doi: 10.3233/JND-210767 ; PubMed Central PMCID: PMC9398077. 35723109 PMC9398077

[46] Pane M, Coratti G, Brogna C, Mazzone ES, Mayhew A, Fanelli L, et al. Upper limb function in Duchenne muscular dystrophy: 24 month longitudinal data. PLoS One. 2018;13(6):e0199223. Epub 20180620. doi: 10.1371/journal.pone.0199223 ; PubMed Central PMCID: PMC6010252. 29924848 PMC6010252

[47] McDonald CM, Gordish-Dressman H, Henricson EK, Duong T, Joyce NC, Jhawar S, et al. Longitudinal pulmonary function testing outcome measures in Duchenne muscular dystrophy: Long-term natural history with and without glucocorticoids. Neuromuscul Disord. 2018;28(11):897–909. Epub 20180829. doi: 10.1016/j.nmd.2018.07.004 . 30336970

