# Supplementary material for: Characterization of patients with Duchenne muscular dystrophy across previously developed health states
Source: PLoS One. 2024 Oct 30;19(10):e0307118. doi: 10.1371/journal.pone.0307118 (PMC11524485; doi:10.1371/journal.pone.0307118)
Supplement: S1 Table — (DOCX) [file pone.0307118.s001.docx]

**S1 Table. Age distribution quantiles across health states for all visits^a^**

| **Health State** | **N Patient** | **N Visit** | **Min** | **1%** | **25%** | **50%** | **75%** | **99%** | **Max** | |
| --- | --- | --- | --- | --- | --- | --- | --- | --- | --- | --- |
| Early Ambulatory | 951 | 3920 | 2.58 | 4.07 | 7.00 | 8.37 | 9.92 | 14.69 | 18.58 | |
| Late Ambulatory | 403 | 1019 | 4.33 | 6.58 | 9.25 | 10.58 | 12.36 | 18.84 | 21.31 | |
| Transfer | 50 | 60 | 3.33 | 5.30 | 10.31 | 12.02 | 12.75 | 16.42 | 16.42 | |
| HTMF, No Ventilator | 82 | 208 | 7.09 | 7.73 | 11.15 | 13.26 | 15.03 | 19.36 | 21.30 | |
| No HTMF, No Ventilator | 15 | 24 | 9.67 | 9.93 | 12.22 | 14.20 | 16.61 | 17.94 | 17.98 | |
| HTMF, Night Ventilator | 17 | 31 | 9.96 | 10.31 | 13.56 | 15.80 | 17.61 | 20.81 | 20.94 | |
| No HTMF, Night Ventilator | 9 | 24 | 13.68 | 13.70 | 15.08 | 16.67 | 18.46 | 20.09 | 20.21 | |
| Full Ventilation | 10 | 20 | 14.35 | 14.39 | 15.96 | 16.92 | 17.68 | 18.42 | 18.45 | |
| **Notes:** | | | | | | | | | |  |
| [a] Some patients may contribute multiple visits within and across health states. | | | | | | | | | |  |
