# Supplementary material for: Characterization of patients with Duchenne muscular dystrophy across previously developed health states
Source: PLoS One. 2024 Oct 30;19(10):e0307118. doi: 10.1371/journal.pone.0307118 (PMC11524485; doi:10.1371/journal.pone.0307118)
Supplement: S2 Table — (DOCX) [file pone.0307118.s002.docx]

**S2 Table. Statistics for ages at first visits per health state per patient**

| **Health State** | **N Patient^a^** | **N Visit^a^** | | **Mean Age** | **SD** | | **Skewness** | | **Kurtosis^b^** | **Min** | | **1%** | | **25%** | **50%** | | **75%** | **99%** | **Max** | | **Time since last visit (yrs)** | | | |
| --- | --- | --- | --- | --- | --- | --- | --- | --- | --- | --- | --- | --- | --- | --- | --- | --- | --- | --- | --- | --- | --- | --- | --- | --- |
| Early Ambulatory | 951 | 987 | | 7.79 | 2.39 | | 0.57 | | 0.40 | 2.58 | | 3.27 | | 5.99 | 7.55 | | 9.34 | 14.23 | 17.97 | | 0.42 | | | |
| Late Ambulatory | 403 | 427 | | 10.57 | 2.56 | | 0.82 | | 1.32 | 4.33 | | 5.70 | | 8.88 | 10.24 | | 11.99 | 17.99 | 21.31 | | 0.61 | | | |
| Transfer | 50 | 50 | | 11.61 | 2.42 | | - 0.59 | | 1.69 | 3.33 | | 4.97 | | 10.27 | 11.67 | | 12.81 | 16.29 | 16.42 | | 1.21 | | | |
| HTMF, No Ventilator | 82 | 89 | | 12.81 | 3.00 | | 0.32 | | - 0.17 | 7.09 | | 7.62 | | 10.51 | 12.56 | | 14.61 | 19.60 | 21.30 | | 0.59 | | | |
| No HTMF, No Ventilator | 15 | 17 | | 14.01 | 2.43 | | - 0.10 | | - 0.91 | 9.67 | | 9.85 | | 11.79 | 13.95 | | 15.83 | 17.86 | 17.98 | | 0.50 | | | |
| HTMF, Night Ventilator | 17 | 20 | | 14.80 | 2.87 | | 0.29 | | - 0.57 | 9.96 | | 10.19 | | 12.64 | 14.57 | | 16.53 | 20.17 | 20.24 | | 0.56 | | | |
| No HTMF, Night Ventilator | 9 | 10 | | 15.87 | 1.83 | | 0.51 | | - 0.87 | 13.68 | | 13.69 | | 14.68 | 15.58 | | 16.70 | 18.92 | 18.97 | | 0.49 | | | |
| Full Ventilation | 10 | 10 | | 16.62 | 1.39 | | - 0.63 | | - 0.92 | 14.35 | | 14.37 | | 15.87 | 16.96 | | 17.71 | 18.27 | 18.32 | | 0.73 | | | |
| **Notes:** | | |  | | |  | |  | | |  | |  | | |  | |  |  |  |  |  |  |  |
| [a] The number of patients and number of visits may differ because single patients may have multiple "first visits" per health state. | | | | | | | | | | | | | | | | | | | | | | | |  |
| [b] Kurtosis is presented as the Excess Kurtosis, i.e., the fourth standardized moment minus 3 to provide a simple comparison to the normal distribution. | | | | | | | | | | | | | | | | | | | | | | | |  |
