# Supplementary material for: Characterization of patients with Duchenne muscular dystrophy across previously developed health states
Source: PLoS One. 2024 Oct 30;19(10):e0307118. doi: 10.1371/journal.pone.0307118 (PMC11524485; doi:10.1371/journal.pone.0307118)
Supplement: S3 Table — (DOCX) [file pone.0307118.s003.docx]

**S3 Table. Summary of characteristics across health states in sensitivity analysis^a-e^**

|  | **Early ambulatory** | **Late ambulatory** | **Transfer** | **HTMF, no ventilation** | **No HTMF, no ventilation** | **HTMF, night-time ventilation** | **No HTMF, night-time ventilation** | **Full-time ventilation** |
| --- | --- | --- | --- | --- | --- | --- | --- | --- |
|  | N = 3,925 | N = 1,025 | N = 39 | N = 208 | N = 24 | N = 31 | N = 24 | N = 20 |
|  | (953 patients) | (406 patients) | (37 patients) | (82 patients) | (15 patients) | (17 patients) | (9 patients) | (10 patients) |
| **Demographics and medications** | | | | | | | | |
| Age (years) | 8.47 ± 0.07 | 10.85 ± 0.13 | 11.70 ± 0.42 | 13.17 ± 0.32 | 14.33 ± 0.62 | 14.94 ± 0.68 | 16.44 ± 0.59 | 16.84 ± 0.37 |
| Height (cm) | 121.06 ± 0.35 | 131.45 ± 0.58 | 142.58 ± 3.16 | 143.56 ± 1.64 | 153.24 ± 4.29 | 151.03 ± 4.20 | 157.50 ± 3.79 | 164.62 ± 2.10 |
| Weight (kg) | 28.02 ± 0.30 | 36.70 ± 0.69 | 49.30 ± 3.13 | 48.76 ± 1.78 | 51.15 ± 3.85 | 56.24 ± 5.00 | 63.89 ± 4.83 | 60.88 ± 6.11 |
| Race |  |  |  |  |  |  |  |  |
| White | 1,545 (85.55%) | 586 (78.98%) | 7 (100.00%) | 177 (88.06%) | 24 (100.00%) | 30 (96.77%) | 24 (100.00%) | 17 (100.00%) |
| Asian | 142 (7.86%) | 42 (5.66%) | 0 (0.00%) | 6 (2.99%) | 0 (0.00%) | 0 (0.00%) | 0 (0.00%) | 0 (0.00%) |
| Black or African American | 19 (1.05%) | 23 (3.10%) | 0 (0.00%) | 1 (0.50%) | 0 (0.00%) | 0 (0.00%) | 0 (0.00%) | 0 (0.00%) |
| Other | 100 (5.54%) | 91 (12.26%) | 0 (0.00%) | 17 (8.46%) | 0 (0.00%) | 1 (3.23%) | 0 (0.00%) | 0 (0.00%) |
| Ethnicity |  |  |  |  |  |  |  |  |
| Hispanic or Latino | 219 (20.02%) | 80 (18.78%) | 0 (0.00%) | 2 (40.00%) | 0 (0.00%) | - | - | - |
| Not Hispanic or Latino | 875 (79.98%) | 346 (81.22%) | 5 (100.00%) | 3 (60.00%) | 1 (100.00%) | - | - | - |
| Steroid use |  |  |  |  |  |  |  |  |
| Deflazacort or prednisone | 3,221 (92.98%) | 940 (97.71%) | 30 (100.00%) | 191 (91.83%) | 19 (79.17%) | 26 (83.87%) | 16 (66.67%) | 2 (10.00%) |
| On daily regimen | 1,980 (66.67%) | 632 (71.49%) | 10 (40.00%) | 134 (70.16%) | 12 (63.16%) | 17 (65.38%) | 14 (87.50%) | 2 (100.00%) |
| Not on steroids | 243 (7.02%) | 22 (2.29%) | 0 (0.00%) | 17 (8.17%) | 5 (20.83%) | 5 (16.13%) | 8 (33.33%) | 18 (90.00%) |
| **DMD measures** | | | | | | | | |
| NSAA total score | 23.69 ± 0.19 | 12.68 ± 0.23 | 3.76 ± 0.31 |  |  |  |  |  |
| NSAA total score IQR | (20.00, 30.00) | (9.00, 16.00) | (2.75, 5.00) |  |  |  |  |  |
| Timed 4-stair climb (s) | 4.50 ± 0.13 | 13.18 ± 0.54 | 18.30 ± - |  |  |  |  |  |
| Health utility index | 0.83 ± 0.02 | 0.77 ± 0.04 | 0.48 ± - |  |  |  |  |  |
| PUL |  |  |  |  |  |  |  |  |
| Total score |  |  |  | 61.30 ± 0.73 | 38.00 ± 1.85 | 53.58 ± 2.73 | 30.99 ± 3.02 | 21.91 ± 3.93 |
| Entry question |  |  |  | 4.55 ± 0.12 | 0.96 ± 0.04 | 3.43 ± 0.27 | 1.00 ± - | 1.00 ± 0.00 |
| HTMF |  |  |  | 2.79 ± 0.05 | 0.89 ± 0.19 | 2.28 ± 0.18 | 0.31 ± 0.12 | 0.24 ± 0.06 |
| Remove lid from container |  |  |  | 0.93 ± 0.02 | 0.74 ± 0.11 | 0.90 ± 0.05 | 0.52 ± 0.13 | 0.13 ± 0.09 |
| Push on the light |  |  |  | 2.61 ± 0.05 | 2.10 ± 0.20 | 2.30 ± 0.17 | 1.50 ± 0.23 | 0.89 ± 0.27 |
| FVC%p (%) | 94.47 ± 0.77 | 89.13 ± 1.33 | 76.75 ± 3.09 | 77.20 ± 1.69 | 69.34 ± 4.55 | 42.34 ± 1.19 | 39.19 ± 1.27 | 20.57 ± 1.63 |
| Left ventricular ejection fraction (%) | 63.91 ± 0.37 | 61.73 ± 0.90 | 64.06 ± - | 56.87 ± 1.36 | 55.10 ± 4.55 | 59.07 ± 48.69 | 46.77 ± 4.91 | 47.70 ± 2.81 |

**Abbreviations:** DMD, Duchenne muscular dystrophy; FVC%p, forced vital capacity percent predicted; HTMF, hand-to-mouth function; NSAA, North Star Ambulatory Assessment; PUL, Performance of Upper Limb; IQR, Interquartile range.

**Notes:**

[a] Data are presented as mean±SE or n (%). To account for correlations with multiple observations per patient, means and standard errors were estimated using generalized estimating equations.

[b] Two outliers, one with height ≤11.8 cm and one with weight ≤3.3 kg, were removed from the calculations.

[c] Percentages were calculated from non-missing data.

[d] Gray cells correspond to unavailable data for a given health state.

[e] In this sensitivity analysis, a visit was classified as ‘able to walk 10m’ if there was a non-missing 10MWR time recorded, or a NSAA walk item (#2) > 0, or a NSAA run item (#17) > 0. A visit was classified as ‘not able to walk 10m’ if there was a missing 10MWR time recorded, NSAA walk item = 0, and a missing or 0 NSAA run item, or a missing 10MWR time recorded, a missing NSAA walk item, and a NSAA run item = 0. All other visits were considered as unclassifiable and excluded from the analysis. This impacts the ambulatory and transfer states, which are defined based on ability to rise from the supine position, walk 10 m, and remain standing.
